# Sustained virologic response improved the long-term health-related quality of life in patients with chronic hepatitis C: a prospective national study in China

**DOI:** 10.1186/s12879-023-08940-3

**Published:** 2024-01-10

**Authors:** Rui Huang, Jia Shang, Hong Chen, Jun Li, Qing Xie, Jiajun Feng, Lai Wei, Huiying Rao

**Affiliations:** 1 Beijing Key Laboratory of Hepatitis C and Immunotherapy for Liver Diseases, Peking University People’s Hospital, Peking University Hepatology Institute, Beijing International Cooperation Base for Science and Technology On NAFLD Diagnosis, No.11 Xizhimen South Street, Beijing, 100044 China; 2 Henan Provincial People’s Hospital, No.7 Weiwu Street, Zhengzhou, 463599 China; 3 First Hospital of Lanzhou University, No.1 Donggang west Street, Lanzhou, 730013 China; 4 First Affiliated Hospital with Nanjing Medical University, No.22 Hankou Street, Nanjing, 210033 China; 5 Medical College, Ruijin Hospital, Shanghai Jiaotong University, No. 573, Xujiahui Street, Shanghai, 200020 China; 6 Department of Marketing, School of Business, Renmin University of China, No. 59 Zhongguancun Avenue, Beijing, 100871 China; 7 Hepatopancreatobiliary Center, Beijing Tsinghua Changgung Hospital, Tsinghua University, No.168, Litang Road, Changping District, Beijing, 102218 China

**Keywords:** Hepatitis C virus (HCV), Health-related quality of life (HRQoL), Sustained virological response (SVR)

## Abstract

**Background:**

To investigate the trends in health-related quality of life (HRQoL) among hepatitis C virus (HCV) patients and to assess the longitudinal impact of antiviral therapy on their well-being.

**Methods:**

In this prospective multicenter observational study in adults with HCV infection, sociodemographic, clinical characteristics and EQ-5D questionnaires were collected. Generalized estimating equation (GEE) models were used to assess the associations between these variables and changes in HRQoL over time.

**Results:**

456 patients were included, with a median age of 46.5 (36.5–57.0) years, of which 262 (57.5%) were males and 44 (9.6%) had cirrhosis. 335 patients (73.5%) receiving antiviral therapy and 61.8% achieved sustained virologic response (SVR). The baseline EQ-5D utility and EQ-VAS were 0.916 ± 0.208 and 80.6 ± 13.0. In multivariable analysis of GEE estimation, achieving SVR24 was positively associated with EQ-5D utility (*p* = 0.000) and EQ-VAS (*p* = 0.000) over time. Age and income were shown to be significant predictors of EQ-5D utility, while gender, age and genotype were associated with EQ-VAS over time.

**Conclusions:**

SVR improved long-term HRQoL in HCV patients in the first few years following viral clearance. Certain sociodemographic factors, such as gender, age, income as well as genotype, significantly influenced long-term changes in patients’ quality of life.

**Trial registration:**

NCT01594554. Registration date: 09/05/2012.

**Supplementary Information:**

The online version contains supplementary material available at 10.1186/s12879-023-08940-3.

## Background

It was estimated that 57.8 million (0·8%) people were living with chronic hepatitis C virus (HCV) infection worldwide [1]. Hepatitis C is responsible for an estimated 399,000 deaths each year, primarily from cirrhosis and hepatocellular carcinoma (HCC) [2]. The disease burden of HCV infection is significant, as it can cause chronic liver disease that can progress over time, leading to significant morbidity, mortality, and healthcare costs. The key milestone in the therapy of hepatitis C was direct-acting antivirals (DAAs) in the 2010s, with rates of sustained virologic response (SVR) above 95% and are generally well-tolerated [3, 4]. Although the global burden of HCV has been on the decline, the impact of hepatitis C in terms of disease burden remains substantial, as evidenced by disability-adjusted life years (DALYs) of 146.2 attributed to HCV-related cirrhosis and 34.9 attributed to HCV-related HCC in the year 2019 [5, 6].

Severe impairment of patient-reported outcomes (PROs) in patients with chronic HCV infection was seen in real-world practices across the world, while fatigue and psychiatric comorbidities were associated with significant PROs impairment in chronic hepatitis C (CHC) patients [7]. Besides, our previous study indicated health-related quality of life (HRQoL) of Chinese CHC patients was also impaired and associated with symptoms of discomfort, cirrhosis and depression [8]. Some studies suggested that utilities improved after DAAs treatment in patients with CHC [9, 10].

Although there are some relevant studies [11, 12], there is currently a lack of prospective longitudinal studies in the real world on HRQoL of hepatitis C patients who have achieved SVR. It is not yet clear which factors will continue to impact the quality of life of HCV patients after virus elimination. Expecting to provide some new evidence for the medical care of HCV patients after SVR, we conducted this prospective longitudinal multicenter study across China to understand the trends of change in HRQoL of Chinese HCV patients after SVR in the real world and the impact of antiviral therapy on them.

## Methods

This study aimed to assess longitudinal impact of antiviral treatment and SVR on HRQoL of patients with CHC, and to identify significant predictors of the HRQoL in these patients.

### Study design and patients

This study is a component of the prospective observational study, CCgenos (ClinicalTrials.gov identifier NCT01594554; BMS study ID AI452-018ST, Registration date: 09/05/2012), which was conducted at 28 university hospitals across China. The cross-sectional phase of the CCgenos study included Han Chinese adults who had recently been diagnosed with chronic HCV infection, characterized by a positive anti-HCV antibody and HCV-RNA test, and who had not undergone prior interferon-based treatment for HCV or hepatitis B virus [13]. Following the cross-sectional study, patients were invited to participate in a 5-year follow-up starting in 2012. Patients who did not provide consent to be followed up, patients for whom the data did not qualify for analysis due to major protocol deviation(s) and patients were formally enrolled into any investigational drug clinical trial after the cross-sectional phase of the CCgenos study were excluded. Patients without complete data about HRQoL were also excluded. No other exclusion criteria were applied for the follow-up phase, and no randomization or protocol-driven treatment was implemented [14]. The study protocols were approved by a central review board (the ethics committee of Peking University People’ s Hospital), the institutional review boards of each participating center, and the China National Human Genetic Resource Management Office (2010). The study adhered to the International Society for Pharmacoepidemiology guidelines for Good Epidemiology Practices and applicable regulatory requirements. All patients provided written informed consent before participating in the study.

### Sociodemographic and clinical characteristics

At enrollment, we collected sociodemographic characteristics such as gender, age, place of residence, marital status, occupation, level of education, and economic status. Residence was recorded according to patients’ current locations, including east, west, south, north and central. The division of China into its eastern, southern, western, northern, and central regions is based on geographical directions. Occupation includes white collar (cadre, health care, teacher and self-employed), blue collar (laborer, farming, service and athlete), unemployed and other (student and others). We also considered various clinical characteristics, including discomfort symptoms such as moderate or severe malaise, decreased appetite, nausea, discomfort in the right hypochondrium, fever, xanthochromia, xanthurenic acid, and other uncomfortable symptoms. Due to the diverse extrahepatic manifestations of CHC, and considering the previous literature reporting that these manifestations might affect the HRQoL [15], we examined extrahepatic manifestations, such as Behcet’s disease, cryoglobulinemia, prediabetes, diabetes, hyperlipidemia, fatty liver, insulin resistance, fibromyalgia, membranoproliferative glomerulonephritis, membranous nephropathy, multiple myeloma, Raynaud’s syndrome, rheumatoid arthritis, Sjögren syndrome, systemic lupus erythematosus, thyroid dysfunction, skin lesions, hypertrophic cardiomyopathy, porphyria cutanea tarda, lichen planus, vasculitis, and pulmonary fibrosis. A detailed table that includes diagnosis, symptoms, sign, lab test and pathology of individual extrahepatic manifestation of CHC was constructed to collect the information above.

Cirrhosis was diagnosed using several criteria, including liver biopsy or the presence of ascites, hepatic encephalopathy, upper gastrointestinal bleeding, or Child-Turcotte-Pugh score > 7, or by any two of the following criteria: radiologic imaging showing nodular liver or splenomegaly, platelet count < 100 × 10^9^/L in the absence of other explanations, liver stiffness > 13kPA, or endoscopic detection of gastroesophageal varices. We also calculated Beck’s depression score, which indicates clinical depression with a score of ≥ 17.

We collected clinical information and performed centralized HCV RNA testing every 6 ± 2 months for untreated patients and every 3 ± 1 months for treated patients. To determine SVR24, we analyzed the HCV RNA results collected at the closest time point occurring 24 weeks after the last dose of treatment, which we refer to as SVR24. According to the guideline of HCV infection by World Health Organizasion [16], the HCV RNA measurements were conducted at a central laboratory (the Peking University People’s Hospital) using the Abbott RealTime HCV assay from Abbott Laboratories in Des Plaines, IL, USA, with a lower limit of detection of 12 IU/ml, which was commercialized and had been widely applied.

### EQ-5D

We evaluated the longitudinal HRQoL of patients every 12 months using an approved Chinese version of the EQ-5D-3 L. This measure has been tested for validity and reliability in populations across mainland China. The EQ-5D-3 L is a straightforward multidimensional assessment tool composed of two parts: the EQ-5D descriptive system and the EQ visual analogue scale (EQ-VAS). The EQ-5D-3 L descriptive system categorized patients’ health status into three levels of severity (no, moderate, or severe problems) across five dimensions (mobility [MO], self-care [SC], usual activities [UA], pain/discomfort [PD], and anxiety/depression [AD]), yielding scores that can be converted into a single EQ-5D utility for health status (with 1 representing full health and 0 representing death). We assigned the EQ-5D utility using a Chinese time trade-off value set (Chinese Time Trade-Off Values for EQ-5D Health States) [17].

### Statistical analysis

We conducted a descriptive analysis of patients’ sociodemographic and clinical information, with categorical variables reported as percentages and continuous variables presented as mean ± standard deviation or median (interquartile range). We applied the Bonferroni correction to conduct multiple hypothesis testing to compare the proportion of patients experiencing moderate or severe problems in each domain of the EQ-5D, as well as the mean EQ-5D utility and EQ-VAS scores in patients with and without SVR24. According to the Bonferroni correction formula *p*×(1/n), where *p* is the original threshold (0.05), and n is the total number of tests (n = 6), a *p* value of < 0.008 was considered statistically significant (Table S2).

To examine the associations between sociodemographic and clinical characteristics and changes in HRQoL over time, we applied generalized estimating equation (GEE) models. Sociodemographic and clinical variables were included into univariable analysis, and variables with significant statistical (*p* < 0.05) and time point (baseline and every year up to 5 years) were included in the multivariate analysis. A *p* value of < 0.05 was considered statistically significant. We performed all analyses using SPSS 23.0 (SPSS Inc., Chicago, IL).

## Results

### Baseline sociodemographic and clinical status

The study enrolled 512 patients, but 18 dropped out during follow-up and 15 were excluded due to protocol violations. An additional 23 patients were excluded because they were unable to complete the intact HRQoL assessment, leaving 456 patients for the final analysis. During the follow-up period, joining other clinical studies related to Hepatitis C, loss to follow-up, withdrawal of informed consent, or missing certain visits can all lead to a reduction in the annual count of enrolled patients [14]. The sociodemographic characteristics of the patients are summarized in Table 1, with a median age of 46.5 years (interquartile range: 36.5–57.0) and 57.5% males.

**Table 1:** Baseline sociodemographic characteristics of participants (n = 456)

Parameter	
**Gender**	
Male	262 (57.5%)
Female	193 (42.3%)
**Age, median (Q1, Q3), years**	46.5(36.5, 57.0)
18–39	134 (29.4%)
40–59	233 (51.1%)
≥60	88 (19.3%)
**Residence**	
East	101 (22.1%)
West	99 (21.7%)
South	87 (19.1%)
North	85 (18.6%)
Central	83 (18.2%)
**Marital status**	
Single	56 (12.3%)
Married	384 (84.2%)
Separated/Divorced/Widowed	15 (3.3%)
**Occupation**	
White collar	134 (29.4%)
Blue collar	183 (40.1%)
Unemployed	66 (14.5%)
Other	72 (15.8%)
**Education**	
Primary School	62 (13.6%)
Junior school	117 (25.7%)
High-school	125 (27.4%)
College graduate	151 (33.1%)
**Monthly family income/per person** ^**a**^	
<2000 RMB	252 (55.3%)
2000–4999 RMB	163 (35.7%)
≥5000 RMB	39 (8.6%)

^a^ 1 RMB = 0.1457 USD.

Table 2 presents the clinical characteristics of patients included in the final analysis. Of these patients, 84.0% reported no discomfort symptoms, such as appetite decrease or nausea. Extrahepatic manifestations were reported by 9.7% of patients, and 9.6% had cirrhosis. Beck’s depression score of more than 17, indicating clinical depression, was observed in 9.7% of patients. The major HCV genotypes were genotype 1 (46.1%) and genotype 2 (25.4%). The baseline log10 HCV RNA was 6.10 (5.34, 6.55) IU/mL. Among the 335 patients (73.5%) who received antiviral therapy, 292 (64.0%) and 282 (61.8%) patients achieved SVR12 and SVR24, respectively, during the follow-up period. The median time between the actual detection of HCV RNA and the detection time of SVR24 is 65.5 (18.2, 57.0) days.

**Table 2:** Clinical characteristics of participants (n = 456)

Parameter		
**Route of HCV acquisition** ^**a**^		
Blood Transfusion	271 (59.4)	
Intravenous drug abuse	17 (3.7)	
Dental treatment	92 (21.1)	
Other	87 (19.1)	
**Number of moderate or severe symptoms of discomfort, n (%)** ^**b**^		
0	383 (84.0%)	
1	56 (12.3%)	
≥2	17 (3.7%)	
**Extrahepatic manifestations, n (%)** ^**c**^		
Yes	45 (9.7%)	
No	411 (90.3%)	
**Cirrhosis, n (%)**		
Yes	44 (9.6%)	
No	412 (90.4%)	
**Beck’s Depression Score, median (Q1, Q3)**		
<17	411 (90.1%)	
≥17	45 (9.7%)	
**HCV Genotype**		
1	210 (46.1%)	
2	116 (25.4%)	
Else	129 (28.3%)	
**Log**_**10**_ **HCV RNA, IU/mL, (Q1, Q3)**	6.10 (5.34, 6.55)	
**Duration of follow-up, median months, (Q1, Q3)**	69.6 (69, 70)	
**Antiviral therapy during follow-up, n (%)**		
Yes	335 (73.5%)	
No	121 (26.5%)	
**Achieving SVR 12 during follow-up, n (%)**		
Yes	292 (64.0%)	
No	164 (36.0%)	
**Achieving SVR 24 during follow-up, n (%)**		
Yes	282 (61.8%)	
No	174 (38.2%)	

^a^ Other route of HCV acquisition: sex, long-term exposure history to HCV patient, tattoo, piercings, intra-exam and treatment, and etc. Patients may report more than one route of HCV acquisition. ^b^ Uncomfortable symptoms: moderate or above malaise, appetite decrease, nausea, discomfort of right hypochondrium, fever, xanthochromia, xanthurenic and other uncomfortable symptoms. ^c^ Extrahepatic manifestation: Behcet’s disease, cryoglobulinemia, prediabetes, diabetes, hyperlipidemia, fatty liver, insulin resistance, fibromyalgia, membranoproliferative glomerulonephritis, membranous nephropathy, multiple myeloma, Raynaud’s syndrome, rheumatoid arthritis, Sjögren syndrome, systemic lupus erythematosus, thyroid dysfunction, skin lesions, hypertrophic cardiomyopathy, porphyria cutanea tarda, lichen planus, vasculitis, pulmonary fibrosis. SVR 12/24, Sustained virologic response 12/24 weeks post treatment

### Health-related quality of life in HCV patients

Table S1 demonstrates the comparability of patients included in the study across sociodemographic and clinical factors each year (see Additional file 1). Table 3 indicates that at baseline, the EQ-5D utility and EQ-VAS were 0.916 ± 0.208 and 80.6 ± 13.0, respectively. Of the patients, 33 (7.2%) and 22 (4.8%) reported moderate or severe problems in the MO and UA domains, respectively, while 101 (22.1%) reported moderate or severe pain or discomfort in the PD domain and 98 (21.5%) reported moderate or severe anxiety or depression in the AD domain. Only 6 (1.3%) of patients reported difficulties in the SC domain.

**Table 3:** Quality of life of included participants

	Baseline (n = 456)		1st year (n = 335)		2nd year (n = 331)		3nd year (n = 302)		4th year (n = 253)		5th year (n = 209)	
	Moderate	Severe	Moderate	Severe	Moderate	Severe	Moderate	Severe	Moderate	Severe	Moderate	Severe
**EQ-5D**												
MO, n (%)	31(6.8)	2(0.4)	19(5.7)	0(0)	17(5.1)	1(0.3)	17(5.6)	1(0.3)	17(6.7)	0(0)	16(7.7)	0(0)
SC, n (%)	5(1.1)	1(0.2)	4(1.2)	0(0)	4(1.2)	0(0)	3(1.0)	0(0)	1(0.4)	0(0)	1(0.5)	0(0)
UA, n (%)	19(4.2)	3(0.6)	18(5.4)	0(0)	14(4.2)	1(0.3)	11(3.6)	0(0)	13(5.1)	0(0)	11(5.3)	0(0)
PD, n (%)	96(21.1)	5(1.1)	57(17.0)	3(0.9)	56(16.9)	3(0.9)	56(18.5)	1(0.3)	36(14.2)	1(0.4)	27(12.9)	1(0.5)
AD, n (%)	96(21.1)	2(0.4)	42(12.5)	1(0.3)	38(11.5)	3(0.9)	31(10.3)	1(0.3)	23(9.1)	0(0)	18(8.6)	1(0.5)
**EQ-5D index, mean ± SD**	0.916 ± 0.208		0.938 ± 0.177		0.936 ± 0.199		0.943 ± 0.153		0.956 ± 0.125		0.949 ± 0.159	
**EQ-5D VAS, mean ± SD**	80.6 ± 13.0		82.1 ± 12.2		83.4 ± 12.7		85.9 ± 12.6		85.8 ± 13.1		86.9 ± 12.1	

^a^ Patients reported some problem (moderate or severe) in individual domain. MO, mobility difficulties; SC, self-care difficulties; UA, usual activities difficulties; PD, pain/discomfort; AD, anxiety/depression

The EQ-5D utility and EQ-VAS increased annually, and the proportion of patients reporting severe discomfort in any of the five dimensions of EQ-5D decreased gradually over time (Table 3). Starting from the first year, both the EQ-5D index and EQ-5D VAS scores of SVR group were significantly higher than those of the No SVR group. However, by the fifth year, this inter-group difference was no longer significant (Table S2).

### Individual domain of EQ-5D stratified by SVR

**Fig. 1:**
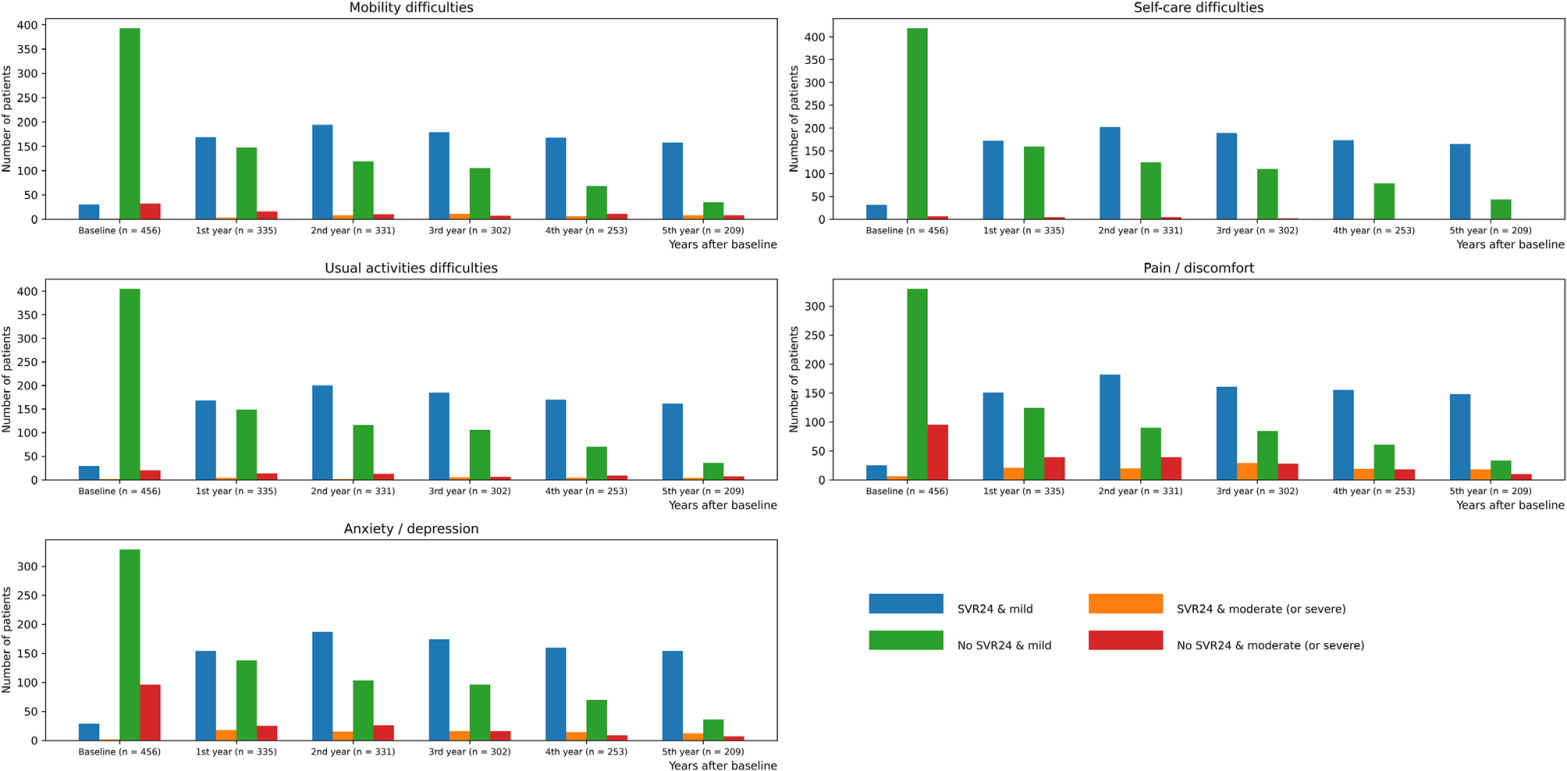
Health-related quality of life domain stratified by SVR or not over time. a Patients reported some problem (moderate or severe) in individual domain. MO, mobility difficulties; SC, self-care difficulties; UA, usual activities difficulties; PD, pain/discomfort; AD, anxiety/depression

Figure 1 illustrates the number of patients who reported moderate or severe problems in individual domains of EQ-5D during follow-up, stratified by SVR status. The numbers between patients with and without SVR became significant over time, particularly in the domains of MO and UA (5th year: MO, 8[4.8%] & 8[18.6%], *p* = 0.032; UA, 4 [2.4%] & 7 [16.3%], *p* = 0.021). Furthermore, a higher proportion of patients without SVR reported pain or discomfort compared to those with SVR (1st year: 21[12.2%] & 39[23.9%], *p* = 0.005; 2nd year: 20[9.9%] & 39 [20.2%], *p* = 0.000; 3rd year: 29[15.3%] & 28 [25.0%], *p* = 0.047; 4th year: 19[10.9%] & 18[22.8%], *p* = 0.027). Table S2 shows this in more detail (see Additional file 1).

### Predictors of longitudinal EQ-5D utility and EQ-VAS score in HCV participants by GEE estimation

GEE estimation was used to explore predictors of longitudinal EQ-5D utility and EQ-VAS. As presented in Table 4 for univariable analysis. Gender, age, residence, marital status, education income, cirrhosis, beck’s depression score and duration of follow-up were found to be significantly associated with EQ-5D utility. Furthermore, achieving SVR24 was positively associated with EQ-5D utility (*β* = 0.049, 95% CI [0.033, 0.065], *p* = 0.000). Gender, age, residence, marital status, education, income, cirrhosis, genotypes and duration of follow-up were significant predictors of EQ-VAS. Meanwhile, SVR24 was found to be a strong predictor of longitudinal EQ-VAS (*β* = 5.933, 95% CI [4.827, 7.040], *p* = 0.000). As presented in Table 5 for multivariable analysis, age, income and SVR24 (*β* = 0.040, 95% CI [0.023, 0.057], *p* = 0.000) were shown to be significant predictors of EQ-5D utility, while gender, age, genotypes and SVR24 (*β* = 5.333, 95% CI [4.204, 6.462], *p* = 0.000) were associated with EQ-VAS over time.

**Table 4:** Predictors of longitudinal EQ-5D index and EQ-VAS score in HCV participants by univariable GEE estimation

Parameter	EQ-5D Index				EQ-VAS Score			
	*β*	SE	*p* value	95% CI	*β*	SE	*p* value	95% CI
**Gender (Ref: Female)**								
Male	0.025	0.012	0.033	0.002, 0.047	3.462	0.928	0.000	1.643, 5.281
**Age (Ref: ≥ 60y)**								
18–39	0.074	0.016	0.000	0.042, 0.105	8.459	1.306	0.000	5.900, 11.019
40–59	0.029	0.017	0.082	-0.004, 0.062	3.166	1.280	0.013	0.658, 5.674
**Residence (Ref: West)**								
Central	0.037	0.018	0.036	0.002, 0.071	1.681	1.397	0.229	-1.058, 4.419
East	0.016	0.019	0.541	-0.025, 0.049	-1.379	1.371	0.314	-4.065, 1.308
North	-0.019	0.022	0.378	-0.062, 0.024	-3.207	1.599	0.045	-6.342, -0.073
South	0.028	0.018	0.118	-0.007, 0.062	1.936	1.352	0.152	-0.714, 4.587
**Marital status (Ref: Single)**								
Separated / Divorced / Widowed	-0.060	0.027	0.029	-0.113, -0.006	-10.961	2.412	0.000	-15.689, -6.233
Married	-0.031	0.016	0.060	-0.063, 0.001	-4.372	1.208	0.000	-6.740, -2.004
**Occupation (Ref: Unemployed)**								
Blue collar	-0.021	0.012	0.085	-0.046, 0.003	-1.306	1.398	0.350	-4.047, 1.435
White Collar	-0.015	0.013	0.234	-0.039, 0.010	-0.781	1.469	0.595	-3.660, 2.099
Other	-0.033	0.018	0.068	-0.068, 0.002	-1.709	1.825	0.349	-5.286, 1.868
**Education (Ref: Primary School)**								
Junior school	0.040	0.026	0.124	-0.011, 0.091	3.261	1.556	0.036	0.211, 6.311
High-school	0.026	0.026	0.316	-0.025, 0.077	3.255	1.586	0.040	0.147, 6.364
College graduate	0.050	0.025	0.047	0.001, 0.100	4.852	1.493	0.001	1.926, 7.778
**Income (Ref: < 2000 RMB)**								
2000–4999 RMB	-0.012	0.013	0.370	-0.037, 0.014	0.424	0.996	0.670	-1.529, 2.377
5000–7999 RMB	0.001	0.021	0.967	-0.041, 0.043	-0.805	2.086	0.700	-4.892, 3.283
8000–9999 RMB	0.064	0.007	0.000	0.049, 0.078	7.329	2.511	0.004	2.408, 12.251
≥10,000 RMB	0.047	0.013	0.001	0.020, 0.074	3.951	3.046	0.195	-2.020, 9.921
**Route of HCV acquisition**								
Blood Transfusion	-0.005	0.009	0.583	-0.022, 0.012	-0.314	0.558	0.574	-1.406, 0.779
Intravenous drug abuse	0.001	0.025	0.972	-0.048, 0.050	-1.936	1.333	0.147	-4.550, 0.678
Dental treatment	0.006	0.009	0.465	-0.011, 0.023	0.864	0.666	0.195	-0.442, 2.170
Other	0.003	0.011	0.813	-0.019, 0.025	0.603	0.642	0.347	-0.655, 1.861
**Symptoms of discomfort (Ref: ≥ 2)**								
0	0.053	0.040	0.194	-0.027, 0.132	4.391	2.638	0.096	-0.779, 9.561
1	0.011	0.044	0.806	-0.076, 0.097	-1.517	2.974	0.610	-7.347, 4.312
**Extrahepatic manifestation (Ref: No)**	-0.022	0.022	0.325	-0.066, 0.022	-2.470	1.755	0.159	-5.909, 0.970
**Cirrhosis (Ref: No)**	-0.061	0.031	0.045	-0.122, -0.001	-5.741	1.640	0.000	-8.955, -2.526
**Beck’s Depression Score (Ref: < 17)**	-0.043	0.017	0.013	-0.077, -0.009	-3.324	2.120	0.117	-7.479, 0.832
**HCV Genotype (Ref: Else)**								
1	-0.028	0.016	0.073	-0.060, 0.003	-4.467	1.220	0.000	-6.857, -2.076
2	-0.016	0.013	0.225	-0.042, 0.010	-2.976	1.122	0.008	-5.175, − 0.777
**Log10 HCV RNA, IU/mL**	0.001	0.005	0.860	-0.009, 0.011	0.001	0.005	0.860	-0.009, 0.011
**SVR24 (Ref: No)**	0.049	0.008	0.000	0.033, 0.065	5.933	0.564	0.000	4.827, 7.040
**Duration of follow-up, month**	-0.019	0.007	0.010	-0.033, -0.004	-1.521	0.531	0.004	-2.562, -0.480

**Table 5:** Predictors of longitudinal EQ-5D index and EQ-VAS score in HCV participants by multivariable GEE estimation

Parameter	EQ-5D Index				EQ-VAS Score			
	*β*	SE	*p* value	95% CI	*β*	SE	*p* value	95% CI
**Gender (Ref: Female)**								
Male					2.042	0.900	0.023	0.278, 3.806
**Age (Ref: ≥ 60y)**								
18–39	0.048	0.016	0.003	0.016, 0.080	5.142	1.319	0.000	2.556, 7.727
40–59	0.015	0.017	0.400	-0.019, 0.049	1.388	1.209	0.251	-0.982, 3.758
**Income (Ref: < 2000 RMB)**								
2000–4999 RMB								
5000–7999 RMB								
8000–9999 RMB	0.047	0.017	0.005	0.014, 0.080				
≥10,000 RMB	0.050	0.017	0.004	0.016, 0.084				
**HCV Genotype (Ref: Else)**								
1					-3.930	1.114	0.000	-6.114, -1.746
2					-1.537	1.034	0.137	-3.565, 0.490
**SVR24 (Ref: No)**	0.040	0.009	0.000	0.023, 0.057	5.333	0.576	0.000	4.204, 6.462

## Discussion

In this prospective longitudinal multicenter study of Chinese patients with CHC, we found that the quality of life of patients in real world was impaired. Certain sociodemographic factors, such as gender, age, income as well as genotype, significantly influenced long-term changes in patients’ quality of life. Notably, HCV clearance had a significant impact on the long-term HRQoL of hepatitis C patients. This study shed light on the longitudinal changes in quality of life of real-world hepatitis C patients and provided a basis for the future medical care of these patients in China.

HCV has been widely reported to have a profound negative impact on HRQoL, work productivity and other PROs [18, 19]. A meta-analysis of 51 clinical studies and 15,053 patients suggested that CHC was associated with a significant impairment in global health status, and the impairment was greater in advanced disease [20]. Sociodemographic and clinical characteristics were found to be associated with HRQoL of HCV patients. For instance, occupation, comorbidities, overweight, and feeling worried about CHC progression affected EQ-5D utility and EQ-VAS scores [21]. In our previous study of 997 Chinese HCV patients, we found significant negative associations between HRQoL and sociodemographic and clinical factors such as moderate or severe symptoms of discomfort, disease profile, and depression [8]. In our real-world study of HCV patients, sociodemographic characteristics including gender, age and income were shown to be significant predictors of HRQoL. Previous studies have indicated disparities in HRQoL of HCV patients in different settings. For example, compared with patients recruited from hospitals, patients recruited from the community setting had lower health utilities [22]. Besides, compared with clinical trial enrollees, patients in real-world practices were less commonly enrolled from high-income population, older, more commonly female, less employed, had more type 2 diabetes, anxiety and clinically overt fatigue but less cirrhosis. Sociodemographic confounders were the main reason of differences in HRQoL of HCV patients in different setting [7]. In longitudinal studies of DAAs treatment, utilities have also been shown to improve after treatment in patients with CHC in various settings. However, community-based patients may still face challenges related to comorbid health and social conditions that are not meaningfully addressed by treatment [9]. The HRQoL of 1,180 participants in a real-world setting from the German Hepatitis C Registry also suggested that roughly half of the patients failed to achieve a clinically important improvement after DAAs therapy [23]. Besides, health conditions and lifestyle also contributed to HRQoL of HCV Patients a lot. For example, community-based patients may face challenges related to comorbid health and social conditions that are not meaningfully addressed by treatment [9]. Treating mental illness by psychoeducation may also contribute to the improved PRO of HCV patients [24]. Another example of the impact of setting on the HRQoL of HCV patients who received DAAs was in people who injected drugs (PWID). HRQoL in PWID patients improved following treatment for HCV infection with elbasvir/grazoprevir, suggesting that eradication of HCV infection with DAAs was associated with improved HRQoL [25]. However, a study on PWID in community settings suggested that DAAs treatment was not associated with longitudinal change in PROs scores [26]. The above content indicated that differences in the quality of life of HCV patients in different environments may be due to sociodemographic factors. The results from clinical trials and clinical studies may not fully explain the factors influencing the quality of life of HCV patients in the real world and the impact of DAA treatment on their quality of life. Therefore, our study provided new insights into this issue in the real world.

It has been reported that HCV clearance improved HRQL and other PROs in HCV patients, regardless of the stage of liver disease. Patients with HCV-related decompensated cirrhosis who were given DAAs showed a clinically significant early and sustained improvement in PROs [27–30]. Marked improvements involving physical, mental, and hepatitis-specific indices in HRQoL of HCV patients with cirrhosis were reported between baseline and SVR12 timepoints. Another study of 1,564 patients, of whom 47% had cirrhosis, suggested that mean PROs scores improved slightly in the overall cohort, and age, baseline mental health issues, and a higher number of health comorbidities were predictors of PROs improvements [31]. Sofosbuvir-based DAAs therapy was associated with a significant improvement in PROs 6 months after treatment end in patients with CHC [32]. However, long-term effects on patient HRQoL after HCV clearance are still lacking. A study assessing the impact of DAAs-mediated HCV clearance on HRQoL from DAAs initiation to 1 year after confirmed SVR24 found that the average SF-8 scores were higher than the Japanese national standard values for bodily pain (BP) and mental health at baseline and for general health at 1-year post-SVR24 [33]. Another real-world study of 1601 HCV patients indicated that the improvements in PROs reported by HCV patients who achieved SVR following DAAs therapy were durable at 12 months post-treatment [12]. The longest period of follow-up in similar studies was nearly 3 years, during which DAAs therapy improved HRQoL significantly in the long term, and the improvement did not correlate with the severity of liver fibrosis [34, 35]. However, another long-term assessment found that although patients with decompensated cirrhosis experienced improvement of HRQoL after DAAs therapy, there was a decline after 2 years [36]. Based on the above research results, we need to conduct longer-term studies to evaluate the long-term impact of SVR on the quality of life of HCV patients in the real world. To our knowledge, our study is currently the longest real-world follow-up study evaluating the impact of SVR on HRQoL in HCV patients. From our research results, HCV patients will experience long-lasting improvements in their quality of life after achieving SVR.

We observed a significant improvement in the anxiety/depression domain following HCV clearance in our study. Previous research also indicated that HCV eradication led to a decrease in anxiety prevalence from 30.4 to 19.1% and depression from 35.2–18.2% [37, 38]. Furthermore, patients with severe mental illness and hepatitis C virus infection can benefit from DAAs treatment [39]. However, in our study, 18 (10.8%) and 12 (7.2%) patients with SVR still reported problems in the pain/discomfort and anxiety/depression domains, respectively. This issue has also been reported in other studies. A meta-analysis revealed that all items of the SF-36 questionnaire improved significantly (p < 0.05) from the pretreatment to post-treatment period, except for bodily pain and role limitations-emotional [10]. Additionally, a real-world study found that mental health remained a concern even after treatment with currently used DAAs medications [40]. We found that starting from the first year, both the EQ-5D index and EQ-5D VAS scores of SVR group were significantly higher than those of the No SVR group. However, by the fifth year, this inter-group difference was no longer significant. This suggests that the impact of SVR on HRQoL of HCV patients may have a time-limited effect, only influencing the first few years after achieving SVR. Whether this effect has a long-term impact remains inconclusive.

The treatment approach itself may also affect the quality of life of CHC patients. It appeared that DAAs therapy did not have a negative impact on, but instead improved, the overall quality of life and psychological state of adult patients with CHC [40, 41]. However, the addition of ribavirin to antiviral regimens can compromise HRQoL indices during antiviral therapy [42]. Patients receiving interferon-based treatment had lower utilities compared to those on interferon-free treatment [20]. Due to the fact that DAAs were only introduced to the Chinese market at the end of 2017, and the formulation and initiation of this research project occurred prior to that, with participation in HCV-related clinical trials and studies being considered as exclusion criteria in the research design. One limitation of our study was that all patients in our study received interferon-containing regimens, which may have underestimated the improvement in quality of life resulting from virus clearance to some extent. Another limitation of this study is that it is an observational study in the real world, without any intervention for HCV patients’ treatment, with a follow-up time of once every six months. The time when the virus was undetectable at the time closest to SVR24 was selected as the time of SVR, so it was not possible to obtain the quality of life at the time of SVR with absolute accuracy. As the timing of HCV RNA testing after cessation of therapy is typically later than SVR24, categorizing patients into SVR24 and No SVR24 groups based on this does not affect the accuracy of grouping. Although, EQ-5D-5 L has been demonstrated to have higher sensitivity, as this is a nationwide, multicenter real-world study with a large sample size, extensive data collection, and a long follow-up period, in order to enhance patient compliance and improve the feasibility of comprehensive data collection, we opted for the relatively simple EQ-5D-3 L instrument. Besides, for the purpose of a more intuitive presentation of the research results, we employed t-tests and chi-squared tests when analyzing the individual domains of EQ-5D stratified by SVR at each time point. However, as a study involving repeated measurements and replicated data, these analytical methods did not account for the elimination of confounding factors. While we addressed the influence of confounding factors such as time through the subsequent Generalized Estimating Equation (GEE) analysis. However, when interpreting the results of this study, it’s important to consider the aforementioned limitations of our research.

## Conclusions

This prospective national study in China indicated that virus clearance improved the long-term HRQoL in patients with CHC in the first few years. Certain sociodemographic factors, such as gender, age, income as well as genotype, significantly influenced long-term changes in patients’ quality of life.

### Electronic supplementary material

Below is the link to the electronic supplementary material.

Supplementary Material 1

## Data Availability

The datasets used and/or analysed during the current study are available from the corresponding author on reasonable request.

## Abbreviations

HCV: Hepatitis C virus
HCC: Hepatocellular carcinoma
DAAs: Direct-acting antivirals
SVR: Sustained virologic response
PROs: Patient-reported outcomes
CHC: Chronic hepatitis C
HRQoL: Health-related quality of life
PWID: People who injected drugs
