# Supplementary material for: Sustained virologic response improved the long-term health-related quality of life in patients with chronic hepatitis C: a prospective national study in China
Source: BMC Infect Dis. 2024 Jan 10;24:72. doi: 10.1186/s12879-023-08940-3 (PMC10782531; doi:10.1186/s12879-023-08940-3)
Supplement: Supplementary file 1 — Supplementary Material 1 [file 12879_2023_8940_MOESM1_ESM.docx]

**Table S1. Baseline sociodemographic and clinical characteristics of participants included in the analysis each year.**

|  | **Baseline**  **(n=456)** | **1^st^ year**  **(n=335)** | **2^nd^ year**  **(n=331)** | **3^nd^ year**  **(n=302)** | **4^th^ year**  **(n=253)** | **5^th^ year**  **(n=209)** |
| --- | --- | --- | --- | --- | --- | --- |
| **Gender** |  |  |  |  |  |  |
| Male | 262 | 187 | 189 | 163 | 140 | 118 |
| Female | 193 | 146 | 139 | 132 | 112 | 90 |
| **Age, median (Q1, Q3), years** | 46.5 (37, 57) | 46.0 (35, 57) | 46.1 (35, 57) | 46.2 (35, 37) | 47.0 (38, 58) | 46.9 (39, 57) |
| 18-39 | 134 | 103 | 103 | 90 | 73 | 56 |
| 40-59 | 233 | 169 | 161 | 147 | 128 | 113 |
| ≥60 | 88 | 61 | 64 | 58 | 51 | 39 |
| **Residence** |  |  |  |  |  | * |
| East | 101 | 85 | 76 | 71 | 68 | 61 |
| West | 99 | 65 | 68 | 67 | 47 | 35 |
| South | 87 | 56 | 64 | 41 | 44 | 54 |
| North | 85 | 66 | 63 | 65 | 61 | 22 |
| Central | 83 | 61 | 57 | 51 | 33 | 37 |
| **Marital status** |  |  |  |  |  |  |
| Single | 56 | 44 | 46 | 41 | 24 | 19 |
| Married | 384 | 279 | 272 | 244 | 221 | 186 |
| Separated/Divorced/Widowed | 15 | 10 | 10 | 10 | 8 | 4 |
| **Occupation** |  |  |  |  |  |  |
| White collar | 134 | 95 | 103 | 91 | 78 | 62 |
| Blue collar | 183 | 137 | 129 | 121 | 105 | 90 |
| Unemployed | 66 | 47 | 41 | 39 | 33 | 23 |
| Other | 72 | 54 | 55 | 44 | 37 | 34 |
| **Education** |  |  |  |  |  |  |
| Primary School | 62 | 41 | 35 | 33 | 31 | 29 |
| Junior school | 117 | 93 | 88 | 81 | 72 | 53 |
| High-school | 125 | 89 | 92 | 79 | 67 | 57 |
| College graduate | 151 | 110 | 113 | 102 | 83 | 70 |
| **Monthly family income/per person ^a^** |  |  |  |  |  |  |
| <2000 RMB | 252 | 194 | 181 | 165 | 136 | 109 |
| 2000-4999 RMB | 163 | 114 | 119 | 107 | 96 | 80 |
| ≥5000 RMB | 39 | 24 | 27 | 22 | 20 | 19 |
| **Number of moderate or severe symptoms of discomfort, n (%) *** |  |  |  |  |  |  |
| 0 | 383 (84.0%) | 281 (83.9%) | 272 (82.2%) | 246 (81.5%) | 213 (84.2%) | 173 (82.8%) |
| 1 | 56 (12.3%) | 38 (11.3%) | 43 (13.0%) | 36 (11.9%) | 29 (11.5%) | 26 (12.4%) |
| ≥2 | 17 (3.7%) | 15 (4.5%) | 14 (4.2%) | 14 (3.7%) | 11 (4.3%) | 10 (4.8%) |
| **Extrahepatic manifestations, n (%) §** |  |  |  |  |  |  |
| Yes | 45 (9.9%) | 34 (10.1%) | 31 (9.7%) | 29 (9.6%) | 24 (9.5%) | 17 (8.1%) |
| No | 411 (90.1%) | 300 (89.6%) | 298 (90.0%) | 267 (88.4%) | 229 (90.5%) | 192 (91.9%) |
| **Cirrhosis, n (%)** |  |  |  |  |  |  |
| Yes | 44 (9.6%) | 31 (9.3%) | 27 (8.2%) | 21 (7.0%) | 21 (8.3%) | 15 (7.2%) |
| No | 412 (90.4%) | 303 (90.4%) | 302 (91.2%) | 275 (91.1%) | 232 (91.7%) | 194 (92.8%) |
| **Beck's Depression Score, median (Q1, Q3)** | 2.71 (0.0, 2.0) | 3.11 (0.0, 3.0) | 3.14 (0.0, 3.0) | 3.29 (0.0, 3.5) | 3.37 (0.0, 3.5) | 2.96 (0.0, 2.0) |
| <17 | 432 | 314 | 308 | 277 | 236 | 197 |
| ≥17 | 23 | 19 | 20 | 18 | 16 | 11 |
| **HCV Genotype** |  |  |  |  |  |  |
| 1 | 210 | 151 | 159 | 149 | 126 | 101 |
| 2 | 116 | 89 | 81 | 77 | 57 | 51 |
| Else | 129 | 93 | 88 | 69 | 69 | 56 |
| **Log_10_ HCV RNA, IU/mL, (Q1, Q3)** | 5.86 (5.36, 6.55) | 5.89 (5.47, 6.58) | 5.80 (5.31, 6.53) | 5.82 (5.34, 6.53) | 5.79 (5.31, 6.53) | 5.80 (5.33, 6.55) |
| **Duration of follow-up, median months, (Q1, Q3)** | 69.6 (69, 70) | 69.8 (69, 70) | 69.6 (69, 70) | 69.7 (67, 70) | 69.7 (69, 70) | 69.7 (69, 70) |
| **Antiviral therapy during follow-up, n (%)** |  |  |  |  |  |  |
| Yes | 355 (77.9%) | 257 (76.7%) | 253 (76.4%) | 230 (76.2%) | 198 (78.3%) | 166 (79.4%) |
| No | 121 (22.1%) | 77 (23.0%) | 76 (23.0%) | 66 (21.9%) | 55 (21.7%) | 43 (20.6%) |
| **Achieving SVR 12 during follow-up, n (%)** |  |  |  |  |  |  |
| Yes | 292 (64.0%) | 225 (67.2%) | 224 (67.7%) | 204 (67.5%) | 177 (70.0%) | 146 (69.9%) |
| No | 164 (36.0%) | 109 (32.5%) | 105 (31.7%) | 92 (30.5%) | 76 (30.0%) | 63 (30.1%) |
| **Achieving SVR 24 during follow-up, n (%)** |  |  |  |  |  |  |
| Yes | 282 (61.8%) | 218 (65.1%) | 217 (65.6%) | 198 (65.6%) | 171 (67.6%) | 142 (67.9%) |
| No | 174 (38.2%) | 116 (34.6%) | 112 (33.8%) | 98 (32.4%) | 82 (32.4%) | 67 (32.1%) |

^*^ *p* < 0.05, compared to patients included at baseline.

**Table S2. Individual domain of EQ-5D stratified by SVR over time.**

|  | **Baseline (n = 456)** | | | **1^st^ year (n = 335)** | | | **2^nd^ year (n = 331)** | | |
| --- | --- | --- | --- | --- | --- | --- | --- | --- | --- |
|  | SVR24  (n = 31) | No SVR24 (n = 425) | *p* value | SVR24  (n = 172) | No SVR24 (n = 163) | *p* value | SVR24  (n = 202) | No SVR24 (n = 129) | *p* value |
| **EQ-5D ^a^** |  |  |  |  |  |  |  |  |  |
| MO, n (%) | 1 (3.2) | 32 (7.5) | 0.373 | 3 (1.7) | 16 (9.8) | 0.002 | 8 (4.0) | 10 (7.8) | 0.138 |
| SC, n (%) | 0 (0.0) | 6 (1.4) | 0.506 | 0 (0.0) | 4 (2.5) | 0.045 | 0 (0.0) | 4 (3.1) | 0.045 |
| UA, n (%) | 2 (6.5) | 20 (4.7) | 0.662 | 4 (2.3) | 14 (8.6) | 0.012 | 2 (1.0) | 13 (10.1) | 0.001 |
| PD, n (%) | 6 (19.4) | 95 (22.4) | 0.699 | 21 (12.2) | 39 (23.9) | 0.005 | 20 (9.9) | 39 (30.2) | 0.000 |
| AD, n (%) | 2 (6.5) | 96 (22.6) | 0.002 | 18 (10.5) | 25 (15.3) | 0.184 | 15 (7.4) | 26 (20.2) | 0.002 |
| **EQ-5D index, mean±SD** | .959 ± .077 | .929 ± .119 | .176 | .970 ± .072 | .928 ± .124 | .000 | .973 ± .073 | .914 ± .141 | .000 |
| **EQ-5D VAS, mean±SD** | 86.2 ± 12.0 | 80.2 ± 13.0 | .013 | 85.5 ± 9.4 | 78.5 ± 13.6 | .000 | 86.5 ± 9.4 | 78.3 ± 15.5 | .000 |
|  | **3^rd^ year (n = 302)** | | | **4^th^ year (n = 253)** | | | **5^th^ year (n = 209)** | | |
|  | SVR24  (n = 190) | No SVR24 (n = 112) | *p* value | SVR24  (n = 174) | No SVR24 (n = 79) | *p* value | SVR24  (n = 166) | No SVR24 (n = 43) | *p* value |
| **EQ-5D ^a^** |  |  |  |  |  |  |  |  |  |
| MO, n (%) | 11 (5.8) | 7 (6.3) | 0.871 | 6 (3.4) | 11 (13.9) | 0.013 | 8 (4.8) | 8 (18.6) | 0.032 |
| SC, n (%) | 1 (0.5) | 2 (1.8) | 0.288 | 1 (0.6) | 0 (0.0) | 0.502 | 1 (0.6) | 0 (0.0) | 0.612 |
| UA, n (%) | 5 (2.6) | 6 (5.4) | 0.223 | 4 (2.3) | 9 (11.4) | 0.018 | 4 (2.4) | 7 (16.3) | 0.021 |
| PD, n (%) | 29 (15.3) | 28 (25.0) | 0.047 | 19 (10.9) | 18 (22.8) | 0.027 | 18 (10.8) | 10 (23.3) | 0.080 |
| AD, n (%) | 16 (8.4) | 16 (14.3) | 0.110 | 14 (8.0) | 9 (11.4) | 0.393 | 12 (7.2) | 7 (16.3) | 0.066 |
| **EQ-5D index, mean±SD** | .960 ± .088 | .939 ± .103 | .055 | .972 ± .077 | .932 ± .115 | .007 | .970 ± .076 | .913 ± .125 | .008 |
| **EQ-5D VAS, mean±SD** | 88.1 ± 11.6 | 82.0 ± 13.3 | .000 | 88.2 ± 11.2 | 80.5 ± 15.4 | .000 | 88.3 ± 10.7 | 81.4 ± 15.7 | .010 |

^a^ Patients reported some problem (moderate or severe) in individual domain. MO, mobility difficulties; SC, self-care difficulties; UA, usual activities difficulties; PD, pain/discomfort; AD, anxiety/depression. *P* value of < 0.008 was considered statistically significant.
